# Development and Implementation of DIALOG+S in the School Setting as a Tool for Promoting Adolescent Mental Well-Being and Resilience in a Post–Armed Conflict Area in Colombia: Exploratory Cluster Randomized Controlled Trial

**DOI:** 10.2196/46757

**Published:** 2023-10-04

**Authors:** Carlos Gómez-Restrepo, María José Sarmiento-Suárez, Magda Alba-Saavedra, Maria Gabriela Calvo-Valderrama, Carlos Javier Rincón-Rodríguez, Victoria Jane Bird, Stefan Priebe, Francois van Loggerenberg

**Affiliations:** 1 Departamento de Epidemiologia Clínica y Bioestadistica Facultad de Medicina Pontificia Universidad Javeriana Bogotá Colombia; 2 Departamento de Psiquiatría y Salud Mental Facultad de Medicina Pontificia Universidad Javeriana Bogotá Colombia; 3 Hospital Universitario San Ignacio Bogotá Colombia; 4 Facultad de Medicina Pontificia Universidad Javeriana Bogotá Colombia; 5 Departamento de Epidemiología Clínica y Bioestadística Facultad de Medicina Pontificia Universidad Javeriana Bogotá Colombia; 6 Unit for Social and Community Psychiatry, Centre for Psychiatry and Mental Health Wolfson Institute of Population Health Queen Mary University of London London United Kingdom; 7 Unit for Social and Community Psychiatry East London NHS Foundation Trust London United Kingdom; 8 Youth Resilience Unit, Centre for Psychiatry and Mental Health Wolfson Institute of Population Health Queen Mary University of London London United Kingdom

**Keywords:** mental health, digital intervention, psychosocial intervention, armed conflict, adolescents, school, DIALOG+, DIALOG+S, mobile phone

## Abstract

**Background:**

Educational settings are ideal for promoting mental well-being and resilience in children. The challenges of the COVID-19 pandemic made evident the important role that teachers and school counselors play in the mental health of their students. Therefore, it is imperative to develop and implement cost-effective interventions that allow them to identify and address mental health problems early, especially in post–armed conflict areas, to reduce the burden of mental disorders in this population.

**Objective:**

This study aimed to adapt an existing patient-focused digital intervention called DIALOG+ from an adult clinical setting to an adolescent educational setting and to assess the feasibility, acceptability, and estimated effect of implementing this intervention as a tool for promoting quality of life, mental well-being, and resilience.

**Methods:**

We conducted an exploratory mixed methods study in 2 public schools in postconflict areas in Tolima, Colombia. This study was conducted in 3 phases. In the adaptation phase, focus groups were conducted with students and teachers to identify changes required in DIALOG+ for it to be used in the school setting. The exploration phase consisted of an exploratory cluster randomized controlled trial. A total of 14 clusters, each with 1 teacher and 5 students, were randomly allocated to either the experimental (DIALOG+S) group or to an active control group (counseling as usual). Teachers in both groups delivered the intervention once a month for 6 months. Through screening scales, information was collected on mental health symptoms, quality of life, self-esteem, resilience, and family functionality before and after the intervention. Finally, the consolidation phase explored the experiences of teachers and students with DIALOG+S using focus group discussions.

**Results:**

The changes suggested by participants in the adaptation phase highlighted the central importance of the school setting in the mental health of adolescents. In the exploratory phase, 70 participants with a mean age of 14.69 (SD 2.13) years were included. Changes observed in the screening scale scores of the intervention group suggest that the DIALOG+S intervention has the potential to improve aspects of mental health, especially quality of life, resilience, and emotional symptoms. The consolidation phase showed that stakeholders felt that using this intervention in the school setting was feasible, acceptable, and an enriching experience that generated changes in the perceived mental health and behavior of participants.

**Conclusions:**

Our results are encouraging and show that the DIALOG+S intervention is feasible and acceptable as a promising opportunity to promote well-being and prevent and identify mental health problems in the school context in a postconflict area in Colombia. Larger, fully powered studies are warranted to properly assess the efficacy and potential impact of the intervention and to refine implementation plans.

**Trial Registration:**

International Standard Randomised Controlled Trial Number (ISRCTN) registry ISRCTN14396374; https://www.isrctn.com/ISRCTN14396374

**International Registered Report Identifier (IRRID):**

RR2-10.2196/40286

## Introduction

### Background

The increasing prevalence of mental health disorders among young people is a public health concern, and actions to address them have become a priority worldwide [1]. Global estimates suggest that 16% of the global burden of disease for people aged between 10 and 24 years is due to neuropsychiatric disorders [1]. Adolescence is a period of life in which key changes in individual development, identity, and interpersonal relationships occur [2], making adolescents more susceptible to mental health problems. This explains why most mental health problems emerge for the first time during adolescence [3].

The onset of the COVID-19 pandemic has been an aggravating factor in the already difficult mental health picture. Several studies have shown that multiple areas of life were impacted by the prolonged school closures and lockdown requirements and that this has increased the burden of mental illness [4-7]. The long-term consequences of these lockdown restrictions for adolescents and their mental health will continue to be felt in the years to come.

Even though there is a clear issue with youth mental health, with pre–COVID-19 findings suggesting that 1 in 7 adolescents had a mental health problem [8], the majority of adolescents who are affected do not have adequate access to support and treatment [4,9]. Multiple governmental, societal, and individual barriers persist and hinder an adequate response to the mental health needs of this population. This is particularly noticeable in countries where inequity, armed conflict, and poverty are experienced. In these contexts, a shortage of mental health professionals, a large mental health treatment gap, a scarcity of funds, and a lack of education and community knowledge concerning mental health issues are some of the barriers that perpetuate the stigma and false beliefs surrounding mental health [10,11].

Mental health promotion and the prevention and early identification of related problems during this life stage are crucial because it is during this period that an individual develops the initial social, emotional, physical, and cognitive tools that allow for a healthy and fulfilling adulthood [2]. When left untreated, mental health disorders have a significant impact on social relationships, academic performance, employment, well-being, and quality of life in the short and long term and possibly intergenerationally, which is even more worrying [3,12].

Therefore, strategies to mitigate and address this problem are urgently required, especially in territories where adolescents face not only the usual challenges of their age and the impact of COVID-19 measures but also the scourge of war, violence, and internal displacement. Exposure to these environments heightens fear, worry, helplessness, anger, and psychological distress among children and adolescents, and international studies have shown that refugees and displaced individuals have a higher risk of presenting mental health disorders such as depression, anxiety, and posttraumatic stress disorder (PTSD) [13-16].

Colombia, with its long history of armed conflict and violence, illustrates these difficulties [17], where adolescent victims of conflict face challenges that have been worsened by the sudden outbreak of the COVID-19 pandemic [18,19]. Between 2019 and 2020, the national poverty rate increased from 35.7% to 42.5%, and as household incomes dropped, other aspects of well-being, such as food security and access to health and education services, also declined [20]. School closures caused an unprecedented crisis in learning and education for the youth of rural municipalities, who, before the pandemic, already faced high levels of illiteracy and school dropout and lower admission to higher education [21,22]. All these are aggravating factors in an already difficult situation. For instance, the 2015 Colombian National Mental Health Survey found a lifetime prevalence of any mental health disorder of 7.2% in adolescents aged between 12 and 17 years [23]. In addition, a significant difference in the prevalence of anxiety and PTSD was found between the displaced population and the nondisplaced population aged between 12 and 17 years. For anxiety, it was 6.6% in the displaced population and 1.8% in the nondisplaced population, and for PTSD, it was 13.2% in the displaced population and 6.6% in the nondisplaced population [24]. According to the Pan American Health Organization, the treatment gap for any mental health disorder in Colombia is 86.1%, the highest of all the countries in the Americas, followed by Guatemala (84.9%) and Mexico (81.4%) [10].

A particularly significant barrier to access to mental health care for Colombian adolescents is the lack of mental health professionals. It has been calculated that in Colombia, there are approximately 1584 psychiatrists, meaning that there are 3 psychiatrists for every 100,000 inhabitants, and health professionals are frequently concentrated in the capital cities and urban regions. Therefore, adolescents in rural areas have limited access to these professionals because of not only costs but also travel and time limitations [25]. One of the biggest challenges to overcome is that the deficit of mental health professionals in rural areas has added to the lack of trained mental health personnel in schools. The scope of the role of schools as places for mental health promotion and the prevention and early identification of mental disorders has increased, because they are key places where adolescents spend most of their time and where most of their social interactions occur. The frequent interaction between teachers and their students creates a trusting bond that can give the educators an opportunity to identify underlying mental health disorders [26,27].

Therefore, strategies to strengthen adolescents’ mental health must be developed in settings that are currently facing these great challenges. After the unprecedented closing of educational institutions during the pandemic, the return to in-person classes means that teachers and educators play an even more important role in promoting mental well-being and resilience in adolescents [22,28,29]. In the Colombian education system, law dictates that there should be 1 school counselor per 500 students [30]. However, this is not followed in all institutions; sometimes, there is only 1 counselor per school and, owing to personnel shortage, the counselor does not necessarily have knowledge or previous experience in mental health [31].

### This Study

To promote a school-based approach to mental health, our study aimed to assess the feasibility of implementing an existing adult patient–centered digitally supported intervention called DIALOG+ through teachers and counselors with Colombian adolescents at schools in Chaparral, Tolima. This is a rural municipality that, after the peace agreements signed by the Colombian government in 2016, is part of the *Program of Development with a Territorial Approach* or *Programas de Desarrollo con Enfoque Territorial* (PDET) [32]. This program aims to prioritize and restore the rights of the inhabitants of the municipalities most affected by the armed conflict. The DIALOG+ intervention aims to help with the identification of mental health conditions and the mobilization of personal and social resources to mitigate the impact of social difficulties and enhance quality of life. This intervention was developed to make the meetings between clinicians and adult patients with mental health issues therapeutically effective [33-36] and has mainly been used in mental health care settings. The adaptation of this tool to adolescents within the school setting is innovative and has not been attempted before. This approach in a postconflict context could help improve the mental health and well-being of Colombian adolescents in vulnerable regions.

## Methods

### DIALOG+ Intervention

DIALOG+ is a digital app–supported and evidence-based intervention created to assist in the interaction between patients with a mental health condition and clinicians. It provides a structured approach to the interaction that takes place in routine clinical meetings and allows patients to actively participate in these meetings [30]. This active participation promotes key aspects, such as self-reflection and expression, which, in turn, empowers patients to improve their mental and social situations through their own actions and by using existing personal and social resources. The intervention is supported by an application that can be installed on multiple electronic devices (eg, tablets or cell phones) and as a progressive web application. During the consultation, DIALOG+ guides the patient to evaluate their satisfaction with 8 quality-of-life areas (mental health, physical health, work, accommodation, leisure activities, friends, relationship with family and partner, and personal safety) and 3 treatment aspects (medication, practical help, and professional appointments). Each item is rated on a scale of 1 to 7, with 1 indicating “totally dissatisfied” and 7 indicating “totally satisfied.” Scores are summarized and displayed on the device, allowing comparisons with scores from preceding meetings. Physicians provide positive feedback on any domain that shows an improvement.

After scoring, the patients are asked whether they would like additional help with each domain. In each meeting, the patient selects up to 3 domains that they have identified as needing assistance with to improve their quality of life. This is discussed through a 4-step solution-focused approach to identify psychosocial resources to intervene in these domains. The first of the 4 steps elicits contextual information about the area under discussion and determines what is working in that area. Step 2 requires the patient to think about the “best case scenario” within that domain and to determine the smallest improvement that can be made to incrementally improve the score on the rating scale. Step 3 invites the patient to reflect on what they and others can do to improve their quality of life. Finally, step 4 summarizes the discussion and makes a list of concrete actions, which are agreed upon and then inputted into the application. Ultimately, the physician and patient together will create an action plan made up of individual action items for each of the discussed areas to be completed before the next session.

### Study Design

To adapt DIALOG+ to the school setting, we conducted a mixed methods study in 2 schools in the Chaparral municipality of the Tolima province. The quantitative component consisted of an exploratory cluster randomized controlled trial where variables concerning mental health, resilience, quality of life, and familiar functionality were measured at baseline and after 6 months of intervention. The qualitative component involved focus groups with teachers and students and a *think-aloud method* [37]. The study design consisted of 3 phases: adaptation, exploration, and consolidation.

During the adaptation phase, we conducted 2 focus groups to understand adaptation strategies to make DIALOG+ appropriate to the Colombian education context. One focus group included 8 adolescents aged between 12 and 18 years, and the other included 8 teachers and counselors. Participants were recruited using convenience sampling. Participants provided informed consent or assent before any focus group participation. Focus groups were led by an experienced facilitator (psychiatrist) and cofacilitator (psychologist) and audio recorded for transcription and analysis. During the session, the existing DIALOG+ tool was presented to participants, and their opinions were used to inform the modifications that were made to the intervention to adapt it to the educational context.

The exploration phase consisted of an exploratory cluster randomized controlled trial to assess the acceptability, feasibility, and estimated effect of applying the DIALOG+S intervention in the school setting. We planned to form 14 clusters, each with 1 teacher and 5 of their students, to achieve a sample of 14 teachers and 70 students aged between 12 and 18 years. Teachers and their students (who together formed a cluster) were randomly allocated to either the experimental (DIALOG+S) group or to an active control group (counseling as usual) by the study coordinator who was blinded to their identities. Teachers, the unit of randomization, were randomized in a 10:4 ratio to the intervention and control groups to maximize our data on the feasibility and acceptability of the intervention while providing some comparison data in the control group to estimate effect. First, teachers were recruited, and then eligible students were identified. Before randomization, each teacher invited 5 adolescents who they considered to be in need of counseling or additional support owing to a personal, family, or social situation that was affecting their performance at school or their well-being. Students who accepted the invitation provided assent, and informed consent was provided by their parents. The experimental intervention was performed using the DIALOG+S tool. Although the Colombian government has programs that guarantee that all public schools in the country have tablets and computers to equip them with these technologies and thus guarantee quality education [38], for the purposes of this study, each teacher was given a tablet with the application and received 90-minute standardized training on its use. The control intervention consisted of 30-minute monthly usual counseling sessions conducted by the teachers without any additional intervention or training. The usual counseling in these schools is based on the professional and teaching experiences of teachers, most of whom have no training in psychology or mental health, and is provided to students who require it according to their performance in school. In our study, we considered this an active control, as these counseling sessions would not ordinarily occur monthly in a structured manner.

Participants from both groups were followed for 6 months. During the study, the students and teachers had six 30 minute-meetings, with 1 meeting conducted every month. During this period, the students only received the intervention to which they were assigned. The control arm students did not have classes or activities with the teachers trained in DIALOG+S, as each teacher who participated in the study only led the specific group of students to which they are assigned. There was no danger of contamination, as the intervention teachers did not interact with the control students.

Teachers who were assigned to the intervention group recorded voice notes, which lasted a maximum of 5 minutes, immediately after each session had been completed, expressing their thoughts and opinions about the implementation process, as well as the difficulties faced during the session, using a *think-aloud method*. Once the voice note was recorded, it was sent to the researchers. Before randomization, the selected adolescents completed a self-administered baseline questionnaire, which was repeated after the 6-month follow-up of the intervention. Data were collected using a standardized paper-based case report form that included questions on sociodemographic characteristics and the following screening scales validated for the Colombian population: the family Adaptability, Partnership, Growth, Affection, and Resolve (APGAR) [39,40] for assessing family function; the Self-Reporting Questionnaire (SRQ) [41] for assessing mental health problems; the 8-item Patient Health Questionnaire (PHQ-8) [42] for measuring depressive symptoms; the 7-item Generalized Anxiety Disorder Scale (GAD-7) [43,44] for measuring anxiety symptoms; the Posttraumatic Stress Disorder Checklist for Diagnostic and Statistical Manual of Mental Disorders, Fifth Edition (PCL-5) [45,46] for measuring PTSD symptoms; the 25-item Connor and Davidson Resilience Scale (CD-RISC-25) [47,48] for assessing resilience; the Rosenberg Scale for assessing self-esteem [49]; and the Manchester Short Assessment of Quality of Life (MANSA) for measuring the quality of life [50]. All questionnaire data were captured into the study database by researchers who were blinded to allocation.

Finally, in the consolidation phase, the voice notes recorded by the teachers using the *think-aloud method* were transcribed, and 2 focus groups were conducted. One focus group included 8 students, and the other included 8 teachers, who participated in the exploratory phase. The focus groups were led by an experienced facilitator (psychiatrist) and cofacilitator (psychologist), audio recorded, and transcribed verbatim. Both groups were asked to discuss their experiences, their opinions on the acceptability and feasibility of using DIALOG+S in the school setting, and the perceived changes in the mental well-being and behavior of the adolescents.

### Participants

Consistent with the exploratory study design, convenience sampling was performed. We conducted the study with 14 teachers and 70 students aged between 12 and 18 years. The recruitment of teachers, counselors, and adolescents took place in 2 public schools in Chaparral, Tolima, through an invitation to participate. Teachers were recruited first, and each teacher invited 5 adolescents who they considered to be in need of counseling or additional support owing to a personal, family, or social situation that was affecting their performance at school or their well-being. Participants were excluded from the study if they intended to change towns or school in the near future or if they could not participate for the full duration of the study. All adolescents who participated provided written assent, and their parents or guardians and teachers were asked to provide informed consent by signing an informed consent form before any study procedures or data collection began. During the consent process, the researchers ensured that participants and parents or guardians were aware of their right to decline participation at any stage of the research and that withdrawing participation would not affect their rights or access to support.

### Ethical Considerations

The study was conducted in accordance with the ethical principles that govern the conduct of research in Colombia and the United Kingdom. It was reviewed and approved by the Pontificia Universidad Javeriana Faculty of Medicine Institutional Research and Ethics Committee (CIE-2020/171) and the Queen Mary University of London Research Ethics Committee (QMERC20.226).

In line with Colombian regulations, before conducting the study procedures, written assent from the adolescents, written consent from their parents or guardians, and written consent from the teachers were obtained. This study was prospectively registered in the ISRCTN registry (ISRCTN14396374).

### Data Management and Analysis

This study used a mixed methods design. Qualitative and quantitative results are presented for a global analysis of the intervention. Qualitative data were analyzed using thematic analysis techniques. Thematic analysis followed the guidelines of Braun and Clark [51]. A pairwise coding phase was conducted by the facilitator and cofacilitator (who led the focus groups) using the NVivo software (version 11; QSR International). Each independently coded the transcripts and categorized the topics. All transcripts were coded line by line. An inductive approach was used to identify the main themes and recurring patterns. Related opinions were grouped into broader categories, which were compared between the different transcripts. The opinions of the participants were summarized using direct quotations, showing the difference in opinions and the consensus and coherence between the groups, where appropriate. An abstract was presented to the research team, and themes and categories were defined. Discrepancies in coding were discussed with the research team and resolved through consensus. Direct illustrative quotations from participants were translated from Spanish to English for this publication.

A descriptive analysis of the quantitative data obtained during the exploratory phase of the study was performed using the R software (version 4.2.0; R Foundation for Statistical Computing). For the description of the sample and scores obtained in the different scales, continuous variables are presented as mean (SD) or median (IQR), depending on whether the distribution is normal or not, and categorical data are reported as absolute values with relative frequencies (percentages). For each intervention group, the difference between the scores obtained on the preintervention and postintervention scales (δ value) was calculated, and based on this δ value, the Mann-Whitney *U* test was applied to compare the 2 arms of the study. The median of the differences in the deltas of the participants in each arm and its IQR were calculated to determine the effect size. As this is an exploratory study, the power to detect differences with statistical significance is limited.

## Results

### Adaptation Phase

The feedback obtained during these initial focus groups suggested that overall, the intervention was perceived as innovative and appealed to both adolescents and teachers. Among the 8 domains dealing with life overall, participants suggested that 5 should be maintained without change, 2 were modified, and 1 was renamed, and a new domain was created to improve clarity and engagement. Table 1 illustrates the changes made in the domains of the DIALOG+ intervention to create the version adapted to school contexts, DIALOG+S.

**Table 1:** Modifications for the adapted version of DIALOG+, DIALOG+S.

Action	Domain and guiding questions in DIALOG+	Domain and guiding questions in DIALOG+S
No change	Mental Health: how satisfied are you with your mental health?	Mental Health: how satisfied are you with your mental health/current emotional situation?
New	N/A^a^	Self-esteem: how satisfied do you feel with yourself?
No change	Physical health: how satisfied are you with your physical health?	Physical health: how satisfied are you with your physical/body health?
Modified	Job situation: how satisfied are you with your job situation?	Academic performance: how satisfied are you with school/classes/homework? School life: how satisfied are you with your relationship with your peers and teachers? Job situation: how satisfied are you with your job, coworkers and boss?
No change	Accommodation: how satisfied are you with the facilities of your house?	Accommodation: how satisfied are you with the facilities of your house? For example, the places where you sleep, eat, take a shower, dress, and perform your daily activities.
No change	Leisure activities: how satisfied are you with your hobbies or the other activities that you practice during your free time?	Leisure activities: how satisfied are you with your hobbies or the other activities that you practice during your free time?
Modified	Partner/family: how satisfied are you with your partner and family?	Family: how satisfied are you with your family relationships? (parents, brothers, sisters, cousins, grandparents) Partner: how satisfied are you with your partner relationship? In case you don’t have one, how satisfied are you with that?
No change	Friendships: how satisfied are you with your friendships?	Friendships: how satisfied are you with your friendships?
Modified	Personal safety: how satisfied are you with your personal safety?	Environmental safety: how satisfied are you with the safety of your neighborhood and municipality?
No change	Medication: how satisfied are you with the medications that you are taking?	Medication: if you are currently in treatment for a disease, how satisfied are you with the medications that you are taking?
Modified	Practical help: how satisfied are you with the practical help that you receive?	Therapeutic/academic support: if you receive support (occupational therapy, psychotherapy, school tutoring), how satisfied are you with them?
Modified	Professional appointments: how satisfied are you with this medical consultation?	Meetings: how satisfied are you with this meeting?

^a^N/A: not applicable.

According to participants, the domains of mental health, physical health, accommodation, leisure activities, and friendships did not require modifications:

It would be good to add that mental health is related to the emotional situation and physical health to the body.

Student 4, aged 12 years

On the domain of mental health, I would also believe that it is important that children can correctly identify the emotions that are most present to them, because from that they can have a better understanding of how their mental health is.

Teacher 8

The one of friendships is very important because it influences a person a lot, even if one says no, it influences too much, I have always been told that one has to have their own defined personality so as not to be influenced by anyone, well let’s say I am one who if he says no, it’s no, but let’s say that there are people who are very flexible in that aspect and allow themselves to be convinced to do things that may not be good.

Student 2, aged 16 years

The *job situation* domain was, not unexpectedly, the one where more modifications were proposed. This domain of the DIALOG+ intervention aims to discuss the work situation of the patient. As the school setting is a vital part of an adolescent’s day-to-day life, participants believed that domains that inquire specifically about school life and environment were required. An *academic performance* domain was recommended, where questions regarding students’ academic performance, homework, and classes were addressed. In addition, a *school life* domain that inquired about students’ satisfaction with their relationship with their school peers and teachers was proposed. Keeping in mind that a considerable percentage of adolescents, particularly in armed conflict–affected regions, are forced to enter the working force for different reasons, the domain *job situation* was retained:

It is important to talk about homework and classes but not everything is studying, it also affects us how teachers and classmates treat us, I would say that they are different things and everything that surrounds us affects our mental health.

Student 6, aged 17 years

I think that it could be treated as different topics and that academic performance not remain only in the study.

Student 3, aged 15 years

The school environment domain would be good because it could help us detect cases of bullying that are not very obvious.

Teacher 3

It is important to keep aside the domain of the employment situation, since there are some students who also work, especially those who work nights and some may do well at work and poorly at school or vice versa.

Teacher 4

Given that adolescence is usually when initial romantic and sexual experiences take place, participants considered it important to split the family or partner domain, leaving one domain pertaining to family related questions and including the questions related to their partner into another domain:

The partner domain is important to talk about separately...here the teachers tell you that having a boyfriend affects your behaviour and grades...that has to do with it...whether or not you have a partner you are always thinking about this issue because of the hormones [laughs].

Student 2, aged 16 years

One realizes that there is a lot of dysfunctional family, that the students do not live with their parents, but with their stepfather, grandmother, so that affects the students because there are many family difficulties of all kinds, including violence...So that topic requires a special space to be able to address it.

Teacher 7

A change in the Spanish translation of the “personal safety” domain to “environmental safety” *(seguridad en el entorno)* was suggested to make it clearer for participants:

Personal security I understand it in two senses, and I don’t know which one it refers to, so I leave here and I feel safe until I get home, or if, for example, I put on a dress and I feel good and safe with that dress or clothes. I think it is necessary to clarify it because it lends itself to confusion.

Student 1, aged 16 years

Finally, an additional “self-esteem” domain was proposed, where the satisfaction with oneself could be assessed, considering the importance of self-esteem in the development of adolescents and in mental well-being:

Security, self-confidence, self-esteem is also important...it seems important to me to talk about it.

Student 5, aged 15 years

Regarding the 3 treatment domains, both teachers and adolescents suggested retaining them with some modifications to two. Another terminology change was proposed for the “practical help” domain, where a modification of the domain name to “therapeutic/academic support” was proposed to avoid confusion about what practical help would entail. This also makes the domain broader, where not only therapeutic support but also any sort of academic counseling can be discussed. Teachers considered it important to retain the “medication” domain, as if a student is receiving pharmacological treatment, follow-up can be done to improve adherence and identify and discuss possible adverse or side effects. Professional appointments were changed to meetings with teachers or counselors to account for the change from a clinical context to a school context:

I think it is important to know what medication the students are taking, although we are not going to medicate, it is good to know what diseases they have and what medications they take...maybe some behaviours are related to side effects like falling asleep in class.

Teacher 2

It would be good for teachers to take into account if you take medication, for example I take for migraine, and I would like to have someone who can tell when you need to take a medication, so that you take it well or to remind you take it.

Student 7, aged 17 years

I understand practical help as things that help the person in a practical, concrete way, like in a clear way...everything that can help me....

Student 3, aged 15 years

I think that practical help could be changed to therapeutic or academic help because it would be if they receive some type of therapy outside of school or if they have some additional help for their academic part, extra classes or reinforcements.

Teacher 3

### Exploratory Phase

Table 2 shows the sociodemographic characteristics of the 70 adolescents included in this study. The average age of the participants was 14.69 (SD 2.13) years, and 56% (39/70) of the participants identified as female. In the Colombian education system, 6th and 9th grades correspond to basic secondary education, and 10th and 11th grades correspond to middle education, which ends with a bachelor’s degree. Students in basic secondary education represented 59% (41/70) of the participants, and those in middle education represented 41% (29/70) of the total sample.

The differences between the groups were related to the number of students in each group and the way in which they were selected by the teachers. As per the study protocol, 20 students were assigned to the control group, and 50 students were assigned to the intervention group. In the control group, there were more male than female students, which is the opposite of the composition of the intervention group.

Of the 70 enrolled student participants, 5 (7%) withdrew from the study (control group: n=1, 1%; intervention group: n=4, 6%) because of their families’ decision to move to other cities or rural areas. During the study, the students in each group received only the assigned intervention. No adverse effects were reported during the study.

Table 3 shows the mean scores of the participants on each of the screening scales before and after the intervention. Before the intervention, both groups had similar scores in scales related to quality of life (MANSA), self-esteem (Rosenberg Scale), and depressive symptomatology (PHQ-8). The resilience scale score (CD-RISC-25) was lower in the intervention group than in the control group. The SRQ, GAD-7, and PCL-5 scores were higher in the control group than in the intervention group. Family APGAR scores were slightly higher in the control group than in the intervention group.

To estimate the effect size of the intervention, differences in medians (δ value) between the scale scores before and after the intervention in each group were calculated. Unsurprisingly, no substantial results were found, as the study was not powered to detect differences, given its exploratory nature. Consequently, any effect sizes and differences observed were interpreted cautiously.

The results from the MANSA showed that the quality-of-life scores increased from the initial to the final assessment for both groups. However, a higher increase was seen in the intervention group. To evaluate resilience, we used the CD-RISC-25. Table 3 shows that the highest mean obtained in the resilience scores was in the control group before the intervention; however, this decreased slightly in the final evaluation. Conversely, the mean of the intervention group was lower initially but increased after the intervention.

As seen in Table 3, the SRQ, PHQ-8, and GAD-7 scores, which measured symptoms of depression and anxiety, showed minor reductions after the intervention in both the control and intervention groups. The reduction in the PHQ-8 score in the intervention group was more substantial. The PCL-5 was used to evaluate probable PTSD; although the mean scores were higher in the control group, our results show that the reduction in scores was small for both groups.

The Rosenberg Scale allowed us to evaluate self-esteem, and the mean scores were similar between the groups throughout the study, with a minimal reduction after the intervention in both groups. The mean scores for the family APGAR, which evaluated family functionality, showed no changes from before to after the intervention in either group.

Regarding the observed effect sizes after the intervention on the MANSA scale, an improvement in the quality-of-life scores was reported after the intervention in both groups, with an effect size of 0.21 in the intervention group. The differences in the medians of the CD-RISC-25 scores showed that the resilience score improved in the intervention group, with an effect size of 1.50. As seen in Table 3, the δ values suggest that the SRQ, PHQ-8, and GAD-7 scores, which measured symptoms of depression and anxiety, were lower after the intervention in both groups. However, the differences in the medians showed only a significant difference in the SRQ scores, with an effect size of −2.0 in the control group and −1.0 in the intervention group. Although no statistically significant findings were found in the comparison between the groups, the observed differences and effect estimates are promising that larger future fully powered randomized studies would provide more conclusive positive results.

**Table 2:** Sociodemographic characteristics of the student participants.

Characteristic		Control (n=20)	Intervention (n=50)	Total (n=70)
Age (years), mean (SD)		14.85 (2.39)	14.62 (2.04)	14.69 (2.13)
**Sex, n (%)**				
	Male	13 (65)	18 (36)	31 (44)
	Female	7 (35)	32 (64)	39 (56)
**School grade, n (%)**				
	6th grade	3 (15)	9 (18)	12 (17)
	7th grade	2 (10)	16 (32)	18 (26)
	8th grade	5 (25)	3 (6)	8 (11)
	9th grade	1 (5)	2 (4)	3 (4)
	10th grade	4 (20)	10 (20)	14 (20)
	11th grade	5 (25)	10 (20)	15 (21)

**Table 3:** Means in preintervention and postintervention scale scores and δ value for comparisons between the groups.

Screening scale	Control (n=19)				Intervention (n=46)		
	Before, mean (SD)	After, mean (SD)	δ (IQR)	Before, mean (SD)		After, mean (SD)	δ (IQR)
MANSA^a^	4.43 (0.98)	4.70 (1.13)	0.42 (−0.12 to 1.00)	4.59 (1.14)		5.13 (0.92)	0.21 (−0.06 to 0.71)
CD-RISC-25^b^	55.26 (17.18)	53.63 (21.16)	−2.00 (−7.00 to 4.50)	50.85 (21.34)		53.72 (20.57)	1.50 (−4.00 to 9.00)
SRQ^c^	9.47 (4.60)	7.58 (5.49)	−2.00 (−3.00 to −1.00)	8.78 (5.00)		7.37 (4.71)	−1.00 (−3.75 to 0.00)
PHQ^d^	9.42 (5.94)	8.26 (6.38)	−2.00 (−5.00 to 0.50)	9.35 (5.53)		7.50 (5.02)	−1.00 (−5.00 to 1.00)
GAD-7^e^	8.58 (4.91)	6.68 (5.94)	−3.00 (−4.50 to 0.50)	7.91 (6.06)		6.33 (4.94)	−1.00 (−3.75 to 1.00)
PCL-5^f^	39.56 (22.79)	37.33 (19.42)	0.50 (−6.75 to 1.75)	26.38 (17.10)		23.75 (15.50)	−4.00 (−7.50 to 5.00)
Rosenberg Scale	28.37 (2.63)	27.26 (3.81)	−1.00 (−2.00 to 0.50)	28.09 (5.06)		27.67 (4.45)	0.00 (−2.00 to 2.00)
Family APGAR^g^	16.58 (5.14)	16.42 (5.15)	0.00 (−2.50 to 2.00)	17.98 (5.52)		17.91 (4.97)	0.00 (−2.00 to 2.00)

^a^MANSA: Manchester Short Assessment of Quality of Life.

^b^CD-RISC-25: 25-item Connor and Davidson Resilience Scale.

^c^SRQ: Self-Reporting Questionnaire.

^d^PHQ-8: 8-item Patient Health Questionnaire.

^e^GAD: 7-item Generalized Anxiety Disorder Scale.

^f^PCL-5: Posttraumatic Stress Disorder Checklist for Diagnostic and Statistical Manual of Mental Disorders, Fifth Edition.

^g^APGAR: Adaptability, Partnership, Growth, Affection, and Resolve.

### Consolidation Phase

#### Overview

To assess the feasibility and acceptability of DIALOG+S in the school context, a thematic analysis was performed based on the experience that teachers and students who completed the 6-month intervention shared in focus groups and the teacher voice notes recorded using the *think-aloud method*. This thematic analysis yielded 3 main themes: reflections on the DIALOG+ application and the use of technology, perceptions of the role of the student-teacher relationship in the implementation of the intervention, and perceived effects on the mental health and behavior of the participating students.

#### DIALOG+S Application and Use of Technology

In general, the use of tablets and a digital application was seen as innovative by all participants. Students particularly highlighted that the ability to visualize their scores using the device allowed them to be aware of their progress and take a more active role during sessions. In addition, they considered that the follow-up of tasks or actions through the 4-step approach was useful for identifying and monitoring improvements or setbacks in their progress:

I liked that in the application each one has a file and it is different from the others, each one has there as their personal space...everyone has different problems and was able to express them and talk about steps to be able to improve that problem or solve it.

Student 2, aged 15 years

I liked that I could see the change in each session, see how the score was increasing and how doing the tasks we agreed on helped me to solve the problems and feel better...this encouraged me to continue with the process.

Student 8, aged 16 years

Teachers believed that the use of technology motivated and engaged students during the meetings and remarked how the structured nature of the application allowed them to guide the sessions with ease:

One day it happened to me that we were going to start the session and the tablet’s battery was discharged so the student said that without the tablet there would be no meeting...they like everything to be recorded there...the tablet is like a representation of what are we doing in the meetings, if they arrive and see it empty-handed, they don’t like it, but technology catches them.

Teacher 1

It is quite useful to have a sequence, a support, to know what steps to follow, how to start, how to organize the session, and the dialogue and what you agree to with the student...is the enriching part of the activity.

Teacher 5

Teachers’ perception of the use of the application was that it was straightforward, and they expressed that no difficulties arose during the conduct of the sessions. Teachers also considered the ability to see the progress or changes of the students through the sessions very appealing and motivating. They felt that having the concrete actions or tasks that were assigned to the student at the end of each session saved in the application made it easier to remember them and conduct proper follow-up in the next session:

I consider that the application was very useful, especially regarding the tasks because...all of the tasks that were set were recorded and could be followed up, and they [students] could see their own process, what they had said and proposed, their goals and they could also evaluate themselves.

Teacher 3

Regarding the application interface, students considered the rating scale ranging from 1 to 7 easy to comprehend; however, teachers suggested that changing the numeric scale to a more visual parameter, such as using emojis or colors, could improve the graphical aspect of the DIALOG+S application. In addition, it was suggested by the teachers that adding a “not applicable” answer to the numeric scale could be useful in cases where a certain domain does not apply to the student or the student does not wish to provide an answer.

Teachers felt that a lack of time could be a factor that impacts the implementation of the intervention in the school setting, as there are many more students in the school than in the study. Participants observed that the first sessions seemed to take longer as they were getting to know each other and the app; however, as the sessions progressed, the time spent was reduced:

When it comes to implementing it, there is always the time factor..., an application of this kind needs time to find out what the student universe is like and what is going through their head.

Teacher 4

The first time you get nervous because you don’t know how the students are going to take the questions, so you go step by step, but later we became more confident and they opened up to tell their experiences and the meetings became more enjoyable and dynamic.

Teacher 2

I would like it to be a little longer so that I can speak more thoroughly and more deeply.

Student 5, aged 14 years

#### Role of the Student-Teacher Relationship

Overall, the perception of the intervention was positive; students reported an interest in continuing the sessions after the end of the study, and teachers considered the experience to be enriching at both personal and professional levels:

There were days when, as a teacher, I felt overburdened, tired and thought about how I was going to sit down and listen to the students, but once the meeting started I felt that it was like a breather, being able to share with others what one thinks and feel, it is a very enriching experience.

Teacher 3

I felt happy when I had the meeting with the teacher because it was a space to relax and she helped me find the solution to my problems. I don’t want it [the meetings] to end, I would even like it to be more frequent.

Student 7, aged 14 years

Both teachers and students felt that the intervention encouraged a closer and more positive interaction between them. They felt that this increased trust and allowed adolescents to talk with ease, which increased across sessions:

It was a very enriching experience. It gives the possibility of getting to know adolescents beyond their role as students, with their own realities, it seems to me that this greatly nourishes the pedagogical work and that one also begins to become aware that it is necessary to approach the boys and girls in other ways.

Teacher 2

In addition, they felt that the creation of spaces where communication beyond academic issues could take place addressed a need that students had. Teachers remarked on the importance of approaching students in these spaces because some are going through complex situations that could easily be overlooked owing to there being only 1 school counselor:

[The intervention] allows a certain closeness, one can get to know information that is not accessible to the naked eye. The students in the first session were not so open, but as it progresses, they realize that it is an open, simple dialogue with a trusted person

Teacher 7

I liked the space with the teacher because it was sharing. Well, we were talking to another person, we were talking for a long time and I was happy, you never talk like that. I started trusting my teacher and I miss it now that the project is over.

Student 3, aged 12 years

Although students highlighted the ability of teachers to guide the sessions, teachers agreed that beyond their teaching experience, they felt that they required more training and education on mental health to improve:

It was difficult for me...because we have no training in how to handle such complex situations that the students experience that have been affecting their mental health for some time...so for me there was always the question: how far can I go? or how far should I go?

Teacher 8

#### Perceived Effects on Mental Health

In general, after the sessions, students reported feeling improvement in certain aspects of their lives, such as trust, self-esteem, and the management of emotions, as well as generally feeling calmer:

It helped me to change my way of thinking, to get rid of negative thoughts, to think that I can achieve what I set my mind to regardless of people’s opinions. I put aside some fears that I had and it helped me to increase my self-esteem.

Student 4, aged 17 years

In the classroom problems with the classmates improved and it was not only me, but several people, yes, [the intervention] gave us steps to have a better coexistence between us. In other words, it helped us to live better. And it gave us alternatives to solve certain conflicts in our families.

Student 1, aged 16 years

Teachers mentioned that after these sessions, they felt more empathetic and less prejudiced toward behaviors of the students. They observed that during the experience, students had fewer disciplinary interventions due to behavioral problems or academic performance, with fewer meetings with the school principal and counseling sessions regarding their discipline or behavior:

...I ended up understanding the context of the students and seeing the roller coaster of emotions that they handle and some very complex situations that they handle in their homes..., I understood them better because one does tend to have prejudices.

Teacher 6

I think that with this experience we saved these students, we made them feel important, knowing that they have a teacher who knows who they are and how their process is going, made them improve their academic performance, they used to spend time in the school principal’s office due to different problems, and during the project we never saw them there again.

Teacher 8

## Discussion

### Principal Findings

Mental health interventions in school contexts have gained more importance in research because of the potential they may have in the promotion of mental well-being as well as in the prevention and early identification of mental health disorders in children and adolescents [52]. The potential role of schools lies not only in the fact that these are the places where children and adolescents spend most of their time but also in the fact that they provide the possibility of overcoming some social and environmental barriers that traditionally impede access to mental health services (ie, social stigma, costs, transportation, and the lack of professionals) [53,54]. Exploring the active role of teachers and counselors in the mental health of their students as a way of addressing overlooked social and personal needs is imperative, especially after the challenges imposed by the COVID-19 pandemic. However, for these interventions to work, it is important not to burden teachers with complex and time-consuming activities [55]. Digital interventions are promising and potentially effective owing to the widespread and increasing use of smartphones, tablets, and computers. Recently, significant efforts have been made in the development and use of different applications in different contexts to help people with their mental health [56]. Our results are consistent with other studies that show that the implementation of digital interventions in different settings is usually well received and particularly engaging for adolescents [57-60].

Our findings also highlight the crucial role that adolescents and stakeholders play in all stages of the development of these interventions to make them appropriate for the settings in which they are going to be used [53,61]. In addition, they reinforce the notion that the development and design of interventions require a constant focus on testing and refining to optimize their acceptability and engagement [62].

The initial phase of this study allowed us to adapt the DIALOG+ digital intervention from an adult clinical setting to an adolescent school setting. Consistent with previous studies [62,63], the changes and domains suggested by participants highlighted the central role of schools in improving the mental health of adolescents. Beyond academic performance, participants wanted to highlight the importance of school coexistence. This is why they suggested including one domain related to academic performance and another related to student life, which allows us to know their satisfaction with their academic situation, school coexistence, and their relationships with their classmates and teachers. These changes combined with the modifications of the domains related to therapeutic and academic support and meetings with teachers or counselors established the adapted version of DIALOG+, DIALOG+S.

Although not powered for inferential analysis, the quantitative data from the exploratory phase showed small improvements in the final assessment of the DIALOG+S group, suggesting that the intervention has the potential to improve aspects of adolescents’ mental health. These aspects include perceived quality of life, resilience, and anxiety symptoms (as seen in the MANSA, CD-RISC-25, and PHQ-8 scores, respectively), where the intervention group showed a greater improvement than the control group. This is suggestive of a possible breakthrough that warrants further exploration in a larger, fully powered study with random allocation.

In DIALOG+ studies, the primary outcome of the intervention is usually an improvement in the MANSA score. Our observed effect size of 0.21 in the intervention group for the MANSA scores was similar to the effect size of 0.20 reported in the original DIALOG trial in a community mental health setting [64]. Subsequent DIALOG+ studies in different clinical settings and populations showed a larger effect size in MANSA scores than that found in the original DIALOG trial [34-37]. Nevertheless, our results are encouraging, considering that this is the first time that the DIALOG+S version has been tested in a school context and that the participants are adolescents living in a postconflict area where socioeconomic conditions have a significant impact on their quality of life.

The fact that the participants were living in a postconflict area could also explain why our results showed lower levels of resilience in both groups compared with other studies in adolescents [65]. Our initial assessment revealed a mean of 50.85 in the intervention group and 55.26 in the control group. These results are similar to those in adolescents who have experienced potentially traumatic events. A mean of 50.2 was reported in Chinese adolescent survivors of a natural disaster, and a mean of 54.7 was reported in displaced Iraqi adolescents [65]. Our PCL-5 scale results showed that the DIALOG+S intervention had no observable impact on PTSD; the complex nature of this condition might require therapeutic interventions by mental health professionals who focus particularly on this disorder [66].

Although the changes in the postintervention scores on the SRQ, GAD-7 and PHQ-8, which measured anxiety and depression symptoms, were small for and similar between both the control and the intervention groups, except for the scores on the PHQ-8, which showed greater improvement in the intervention group, these should not be dismissed. These results are supported by the perceived changes that participants talked about in the qualitative data obtained during the consolidation phase, where students stated that after the meetings with their teachers, they felt more in control of their emotions, perceived less anxiety and improvement in their overall self-esteem, and thought that the intervention gave them tools to solve problems. In addition, teachers noticed improvements in students’ behaviors, which was reflected in fewer disciplinary actions due to academic or behavioral issues and, encouragingly, fewer visits to the school principal’s office and counselor.

The consolidation phase confirmed the acceptability of the intervention among both adolescents and teachers. Our acceptability thresholds were met in terms of completion rates, with only 5 (7%) of the 70 students withdrawing from the study because of moving to other cities and none withdrawing because of reasons related to the intervention. The exploratory phase was completed without major difficulties, suggesting that the 90-minute standardized training provided to the teachers was sufficient for them to understand the functioning of the DIALOG+S application. It was not a given that the adapted application would be successfully delivered by teachers or well received by students in schools. This is an important finding and may initiate a route for early mental health intervention through a digital application in the school setting without overburdening educators. Participants’ reflections on the constraints that emerged during the exploratory phase are very important and require careful consideration because they are key factors for the possible implementation of the DIALOG+S intervention. The teachers first underlined the important impact that providing additional education on mental health to them can have in terms of making them feel more confident and equipping them with more tools to understand and support their students. Given the low number of school counselors, providing teachers with training in the use of this application and resources for learning such as a web-based diploma on school mental health as developed by researchers [67] could give them the skills and confidence to intervene in students with difficulties. This means that not only will the student be heard, but teachers can also identify risky behaviors or possible mental health disorders and refer the student to the counselor, who may, in turn, refer the student to mental health care if necessary. Other studies have shown that most teachers are willing to learn about and be involved in targeted mental health education programs, and many express their frustration and urgency in their need for additional training and support for dealing with the mental health problems of their students [68-70]. Evidence suggests that promoting mental health literacy among teachers significantly increases not only their knowledge of mental health issues but also their motivation to help students and provides them with confidence in delivering interventions. Unsurprisingly, these actions show changes in teachers’ beliefs and reduce perceived stigma [68-70].

The lack of time in the school settings is also a relevant factor. Participants noted that the initial sessions seemed to be longer, as they were getting to know each other, but once they became more familiar with the application as the sessions progressed, less time was required to deliver the DIALOG+S intervention. It is important to note that for these interventions to work, their use should not overburden the students or teachers [62]. Rather than being overburdened, the time spent in the meetings was perceived by students and teachers as an opportunity to strengthen the student-teacher relationship, improve trust, judge less, and get to know each other better. These meetings were also considered a positive change in the usual routine of teachers and students to focus less on the academic and more on the personal aspects.

The feedback obtained during the consolidation phase showed that stakeholders considered the use of this intervention in the school setting feasible. They thought that it was an enriching experience that made teachers understand their students better and adolescents feel closer to their teachers, with a subjective improvement in certain aspects of their lives.

### Limitations

One of the main limitations of our study is the small sample of participants in the exploratory phase. Future studies with larger sample are needed to properly evaluate the effectiveness of the intervention. Teachers chose students who they considered to be in need of additional help because of known distress or problems; however, this may have led to the underrepresentation of students who might not have verbalized their needs but were still struggling with mental health problems and those who were not actively asking for help but may have required it nonetheless. Not selecting the sample based on a low quality of life or mental distress means that it would be harder to show an improvement or change in the sample. In addition, an active control could mask the true effect of the intervention, especially because the standard of care is not monthly counseling sessions. It is important to note that teachers assigned to the control group did not receive any DIALOG+S training, which their counterparts in the intervention group did receive, and this training could have empowered them to use a new problem-solving strategy.

### Conclusions

We explored the potential of implementing an app-based psychosocial intervention to improve adolescents’ quality of life and mental health in the school context in a postconflict area. Our results suggest that the DIALOG+S intervention for the school setting presents a promising opportunity to promote mental well-being and identify and reduce the risk of mental health problems. Our experience is encouraging, as DIALOG+S was perceived as acceptable and usable. A fully powered trial is recommended to assess the efficacy of DIALOG+S in the school setting as a promising intervention for improving the quality of life, resilience, and mental health of adolescents.

## Appendix

Multimedia Appendix 1 CONSORT-eHEALTH checklist (V 1.6.1).

## Abbreviations

APGAR: Adaptability, Partnership, Growth, Affection, and Resolve
CD-RISC-25: 25-item Connor and Davidson Resilience Scale
GAD-7: 7-item Generalized Anxiety Disorder Scale
MANSA: Manchester Short Assessment of Quality of Life
PCL-5: Posttraumatic Stress Disorder Checklist for Diagnostic and Statistical Manual of Mental Disorders, Fifth Edition
PDET: Programas de Desarrollo con Enfoque Territorial (Program of Development with a Territorial Approach)
PHQ-8: 8-item Patient Health Questionnaire
PTSD: posttraumatic stress disorder
SRQ: Self-Reporting Questionnaire

## Data Availability

The data sets used and analyzed during this study are available from the Colombian principal investigator upon reasonable request.
